# Differential Distributions: A refined methodology to indirect reference interval estimation by including Patient's health status according to associated ICD-10 codes

**DOI:** 10.1016/j.plabm.2025.e00492

**Published:** 2025-07-09

**Authors:** David Schär, Tobias U. Blatter, Harald Witte, Jivko Stoyanov, Martin Hersberger, Christos T. Nakas, Alexander B. Leichtle

**Affiliations:** a University Institute of Clinical Chemistry Inselspital - Bern University Hospital and University of Bern, Switzerland; b Graduate School for Health Sciences (GHS) University of Bern, Switzerland; c Swiss Paraplegic Research, Nottwil, Switzerland; d Institute of Social and Preventive Medicine - University of Bern, Switzerland; e Division of Clinical Chemistry and Biochemistry, Children's Research Center, University Children's Hospital Zurich and University of Zurich, Zurich, Switzerland; f Laboratory of Biometry, University of Thessaly, Greece; g Center for Artificial Intelligence in Medicine (CAIM) University of Bern, Switzerland; h Cantonal Hospital Baden, Baden AG, Switzerland

**Keywords:** Machine Learning, Laboratory Medicine, Clinical diagnostics, Reference intervals, Personalized Medicine

## Abstract

**Background:**

Traditional methods for estimating reference intervals (RIs) using patient's blood test results from the clinical routine, typically remove outliers without considering the nuanced health statuses of patients. This removes a vast majority of test results for reference interval estimation without considering the actual health status of the patient.

**Methods:**

We introduce the Differential Distribution Method (DDM) which uses laboratory routine data coded with ICD-10 to approximate an underlying non-diseased age and sex stratified population from mixed clinical data. By removing test results that stem from subpopulations significantly different from the general population, reference intervals can be generated stratified by sex and age, taking into account the associated health conditions of the patients as derived by the ICD-10 coding system.

**Results:**

Applying the DDM to blood plasma potassium levels demonstrated its ability to adjust RIs dynamically across different patient groups. The method effectively differentiated RIs in a decade-based stratification, showing significant variability and tighter confidence intervals, particularly in older (above 60 years old) adults. The RIs were slightly wider with advancing age in both males and females, while their standard deviation was reduced by removing large portions of test results differing significantly, grouped by either their individual ICD-10 code or clusters of ICD-10 codes.

**Conclusions:**

This DDM data mining approach offers a robust framework for RI inference by generating adjusted RIs that incorporate clinical nuances reflected in ICD-10 codes. This approach not only enhances the accuracy of patient diagnostics but also facilitates the identification of potential multimorbidities affecting laboratory results.

## Highlights

• Introducing a novel reference interval inference approach incorporating ICD-10 coding.
• The ICD-10 codes are included in the inference using natural language processing.
• The resulting reference intervals are adjusted by their age, sex and, their health status.
• These reference intervals provide helpful substitutes for elderly or multimorbid patients.

## 1 Background

In clinical diagnostics, accurately determining reference intervals (RIs) is crucial for the correct interpretation of laboratory tests. Traditionally the routine testing of serum and plasma electrolytes, as well as common markers for glomerular filtration rate, such as creatinine and cystatin c, help physicians to estimate patients' renal function [1,2]. The importance of such surveillance increases with older and/or multimorbid patients where these tests are important biomarkers for monitoring acute renal conditions and ensuring appropriate medication management. Stratification according to variables such as “age” and “sex” influence the position and width of the reference interval amongst the test measurements and should offer a reliable source for comparison and evaluation of an individual's test outcome. Current methodologies primarily focus on direct or indirect approaches for RI determination. Direct methods involve recruiting healthy individuals to form a reference group, which is both time-consuming and resource-intensive [3,4]. The rather cumbersome effort of recruiting said non-diseased population *a priori* to infer RIs can be circumvented with indirect approaches. Indirect methods, on the other hand, utilize existing laboratory data from routine patient care but often fail to accurately segregate healthy from “unhealthy” individuals, especially when only demographic factors like age and sex are considered [5,6]. Patients in a clinical setting often present a broad variety of health conditions, a fact that is reflected even in stratified reference populations. This directly influences the RIs, which are estimated from these reference populations. This results in RIs that may not be truly representative of the intended patient population, particularly in settings with high rates of multimorbidity such as intensive care units [7,8]. Consequently, valuable patient data are *a priori* excluded per stratum. This is concerning for elderly patients, as prevalence and comorbidities of diseases are directly linked with age [9]. For elderly patients, a clear differentiation between healthy and non-conspicuous test results is often not achievable with current RIs [10]. The influence of the physiological change due to the patients' health can be significant and should be considered in RI estimation efforts. The Differential Distribution Method addresses these challenges by incorporating the tenth edition of the International Classification of Diseases (ICD-10) coded data to filter and analyze patient populations more precisely [11]. Combinations of such ICD-10 codes allow the representation of a nuanced picture of the patients' health status, including potential comorbidities [12]. By aligning the RI estimation process more closely with the specific health statuses and demographic characteristics of patients, the DDM aims to produce more clinically relevant and personalized RIs. This data mining approach is particularly pertinent in the context of an aging population and the increasing prevalence of chronic diseases, where traditional RI methods may not suffice.

## 2 Methods

### 2.1 Aim

This study introduces the “Differential Distribution Method” (DDM), an approach that utilizes ICD-10 codes associated with each patients’ laboratory measurements. The DDM filters out test results that significantly deviate from the overall distribution within specific age and sex strata, thus generating a health-adjusted reference population. Potassium is chosen as the primary analyte for this study, due to its relevance, frequent measurement in clinical diagnostics and availability in large data sets. The DDM is applicable for analytes for which a large dataset (e.g. > 100000 data points) is available, as this ensures sufficient sample sizes for all patient stata to enable robust and stratified RI estimation. We showcase how the DDM enables age- and sex-stratified “Differential Distributions” (DD), which inform the creation of tailored plasma potassium RIs that incorporate the health conditions reflected in the dataset.

### 2.2 Study population

The retrospective dataset used for this study consisted of anonymized laboratory test results of blood plasma potassium levels obtained from adult patients, aged 20–89 years, treated at the University Hospital Bern, Inselspital. The test results were collected from hospital inpatients between January 2014 and December 2022, who provided informed consent for their data to be used in research. The dataset conforms to the Swiss Personalized Health Network semantic framework [13] and has been described elsewhere in detail [14]. Lithium heparin (LiHep)-Potassium tests (LOINC 2823-3) were performed using an ion-selective electrode (Global Medical Device Nomenclature code 52892) on the Cobas® system (Cobas®8000 series; Roche Diagnostics GmbH), following calibration and maintenance to ensure analytical stability (as per ISO 17025:2017 [15]). Other potassium measurements from the blood gas device, such as Potassium [Moles/volume] in whole blood (LOINC 6298-4), Potassium [Moles/volume] in Arterial blood (LOINC 32713-0), Potassium [Moles/volume] in Mixed venous blood (LOINC 41656-0) were excluded from the analysis. An ethical waiver has been granted by the Bern cantonal ethics committee for use of anonymized data (Req-2020-00630). To minimize the influence of repeated testing, only the first measurement per administrative case was included during data extraction, as practiced in other RI studies [6,16,17]. The patients’ potassium test results were extracted together with associated demographic information such as age (in years) and sex, up to five medical diagnoses in the ICD-10-GM (German Modification) encoding, and details regarding the creation of the measurement (i.e. (pre-)analytical factors). ICD-10 codes were organized into their categories (represented by three-letter codes) to prevent excessive dispersion for the analysis. The data has been pre-processed by removal of data with erroneous ICD-10 codes or data labeled with a negative age prior to the analysis. Furthermore, negative, not-available or biologically impossible test results were also removed.

### 2.3 Statistical analysis

#### 2.3.1 Differential Distribution Method

The Differential Distribution Method (DDM) consists of three steps: A patient factor stratification, a statistical testing, and an RI estimation step. First, the extracted dataset for a specific analyte is stratified by sex (“male” and “female”) and by age, with age ranges of 10 years (20–29, 30–39, …, 70–79, and 80–89). The dataset is separated into data slices for all given combinations of the factors sex and age range and a total distribution covering all values (“Global Distribution”, GD) is created. Associated with the test results in a slice are a variety of unique diagnoses in ICD-10-GM code format (three letter code). In a second step, test results are grouped by each individual ICD-10 code mentioned, and subjected to significance testing against the GD, to assess whether the distributions have notable differences at a prespecified significance level alpha (*α*). Only ICD-10-GM diagnosis subsets with a sample size of more than 5 are considered for two-sided significance testing, however, remain in the overall dataset as this would potentially remove them from other subsets as valid data points. The Student's t-test is used for normally distributed reference values and the Mann-Whitney-U test when normality assumptions are not satisfied. A significance level α = 0.05 is initially chosen, with false discovery rate (FDR) adjustment using the Storey-Tibshirani procedure [18]. Extending on the concept of p-values, the FDR procedure implements q-values which consider the expected proportion of falsely rejected null hypotheses among all rejected hypotheses. The outcome of the hypothesis testing for all ICD-10-GM codes is stored in the form of a table for each slice, containing information on population size (*n*), sample mean ($\tilde{x}$) and sample standard deviation (*s*), p-value, and q-value for each ICD-10-GM code. For a given significance level, the relevant entries in the ICD-10-GM code table can be used to identify test results with a distribution significantly different from the GD. To create the DD, all test results associated with these significantly different ICD-10-GM codes are removed from the GD, resulting in a distribution which contains only test results with associated ICD-10-GM diagnoses that are under the assumption of contributing non-conspicuous test results for each age and sex slice.

#### 2.3.2 ICD-10-GM code clustering

**Fig. 1:**
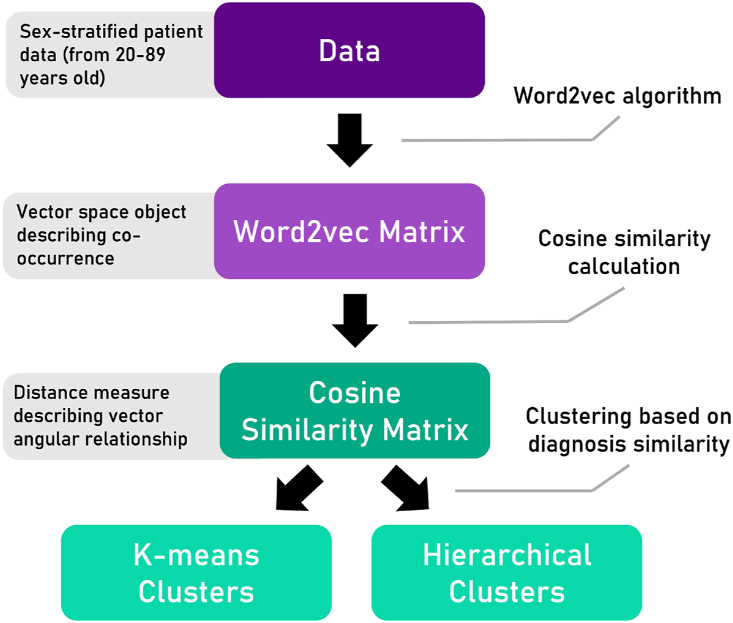
Workflow for Clustering ICD-10-GM Diagnosis Using Natural Language Processing. The process is based on laboratory data that is stratified by analyte and sex. The natural language processing algorithm Word2vec is employed to transform N diagnoses into an N-dimensional vector space. Each diagnosis is represented as a vector, and the angular relationship between these vectors is quantified using cosine similarity.

The DDM also includes a step of clustering ICD-10-GM codes to get a clear understanding of the co-occurrences of ICD-10-GM codes associated with test measurements presented in the routine data. Based on the clustering of ICD-10-GM codes, test results are grouped and the resulting groups are removed from the global distribution if they are statistically different by the aforementioned significance testing after the sex and age stratification step in the DDM. By partitioning values into groups based on co-occurrence of their ICD-10-GM codes, said groups may constitute a more accurate representation of common comorbidities found in a general clinical setting and enhance statistical analysis by increasing their respective population size. With the use of the natural language processing (NLP) technique Word2vec ICD-10-GM codes are partitioned into clusters by obtaining a similarity measure between words (Fig. 1).

In this case, the words are the strings of concatenated ICD-10-GM codes [19]. This NLP technique consists of a shallow neural network able to model *N* words as a *N*-dimensional vector space object based on the frequency words occur together in a text [20]. Each word is represented as a single semantic embedding vector in this space and the angular relationship of two vectors may be quantified as a distance measure using cosine similarity [21]. A *N x N* similarity matrix is drawn containing the pairwise cosine similarity between all ICD-10-GM codes of the slice. Inverting the similarity matrix serves as a distance metric enabling diagnoses to be partitioned into clusters using either k-means clustering or hierarchical clustering [22]. After clustering the ICD-10-GM codes, the statistical testing can be carried out as described before. Finally, in order to create the “DD with Clustering”, all test results associated with these significantly different ICD-10-GM code clusterings are removed from the GD.

#### 2.3.3 Reference interval inference

The created DDs serve as a basis for an iterative method for RI estimation [23]. By iteratively removing proportions of the distribution based on deviation from the mean, this method is robust for approximating the underlying main Gaussian mode when faced with an uneven distribution of extreme values (often labeled as outliers). As outliers are progressively excluded through this process, additional outlier removal steps are not required. The two-sided 95 % RI is defined by the lower and upper bounds and can be inferred by estimating the X_2.5th_ and X_97.5th_ percentiles from the appropriate subpopulation. An estimate of precision can be obtained by calculating the 90 % confidence intervals (CIs) for the percentiles based on their standard error [24]. To compare estimated RIs, the difference between the bounds of the DD RIs and the bound of the GD RIs is calculated. If the bounds of the DDs RIs lie outside the CIs of the GD RI bounds, these RI offer an adjusted estimate considering the health status of the patients. If an overlap between the CIs occurs, the inferred RIs should be further evaluated, as they potentially do not offer a better estimate than RI estimated from the stratified GDs.

#### 2.3.4 Web application

**Fig. 2:**
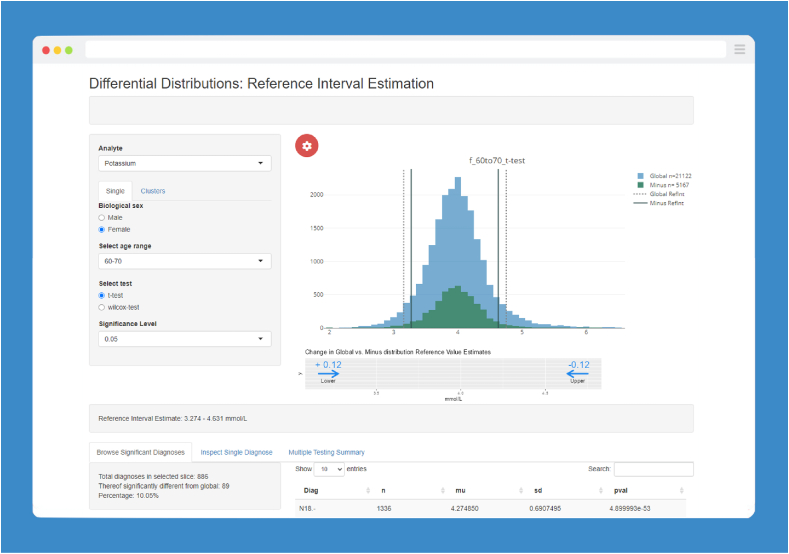
User interface of the Differential Distribution Method (DDM) R Shiny app. The left panel provides user input options for the reference interval estimation process to select variables such as sex, age range, hypothesis testing parameters and significance level thresholds. The main plot area (right) shows histograms representing the Global Distribution (GD) and the Differential Distribution (DD) of selected laboratory measurements. Reference interval estimates are depicted as vertical lines for both distributions. The lower plot section features two directional arrows that represent the magnitude and direction of changes in the reference limits, comparing those estimated from the GD to those from the DD.

In order to fully automate the statistical analysis presented here, a web-based application (“app”) for interactive data analysis was devised and developed. The R Shiny Web application framework (Version 1.7.4) from R Studio was used with R version 4.1.2 [[25], [26]]. The statistical analysis can be run and the subsequent results can be viewed interactively within the app. The data analysis can be viewed in the graphical user interface (GUI) of the app (Fig. 2).

Within the app, for each combination of age and 10-year age range a pre-calculated table is loaded into the app, which allows an interactive analysis by the user. Depending on the user's input, the app will show which ICD-10 diagnoses were significantly different and removed from the GD. The user can search for a significant ICD-10 coded diagnosis, look at detected clusters of ICD-10 codes and inspect the adjusted RI estimated in the analysis. A visual plot is generated for the GD and DD. The distributions are used on the server side of the application to calculate the RIs, which are ultimately shown in the plot as vertical lines.

## 3 Results

### 3.1 Characteristics of the study population

**Fig. 3:**
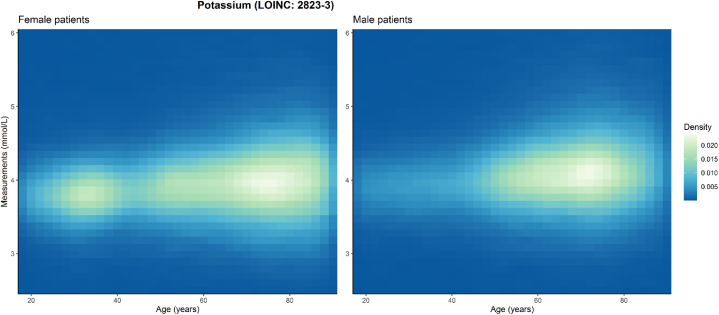
Heatmap of all potassium measurements stratified by age and sex (left: female, right: male). The color intensity represents the density of available potassium measurement for each age year (x-axis) and the respective potassium level in mmol/L (y-axis).

The data used for this study consisted of 289′368 test results of plasma potassium from adult inpatients, spanning the ages of 20–89 years. Among the studied patients, the mean age was 58.6 ± 23.4 years, with 45.2 % women and 54.8 % men from a Swiss adult population. Female adults had a mean age of 57.9 ± 24.6 years, with a median age of 63 years (IQR: 39–78 years), and male adults had a mean age of 59.1 ± 22.4 years, with a median age of 64 years (IQR: 50–75 years). The median potassium levels among the women and men in the study population were 4.0 mmol/L (IQR: 3.7–4.2 mmol/L) and 4.1 mmol/L (IQR: 3.8–4.4 mmol/L), respectively (Fig. 3). An increase in the overall variance of the potassium measurements with age was observed for both female and male study participants.

### 3.2 Potassium differential distribution

The DDM was used to create the DDs from the total potassium data set, stratified by age and sex. To substantiate the method's effectiveness, a detailed analysis of the results from a specific patient stratum is presented. The slice for males, ages 60–69, was initially constituted of 37210 measurements. Nine percent of test values were excluded from the GD to create the DD consisting of 33829 test values. Examination of the major subsets of ICD-10-GM codes (Top 20) showed a diverse range of ICD-10-GM codes (Table 1). To control the FDR, q-values provide adjusted significance levels. Codes referring to renal diseases (see N17, N18), systemic diseases (see E11, I10, I50, and I70), as well codes related to conditions requiring transplantation (T86, Z94) or therapeutic interventions (K35) were all found to have significantly differing test results relative to the GD and were therefore removed with the DDM. The variety of diagnoses observed in this group is also evident in the other slices.

**Table 1:** Key ICD-10-GM Codes Impacting Potassium Test Results in Male Patients Aged 60–69. The table lists the top 20 ICD-10-GM codes significantly impacting potassium test results, differentiated from the GD based on t-tests (α = 0.05). It includes sample size (n), sample mean ($\tilde{x}$), and sample standard deviation (s) for each diagnosis. Q-values, representing adjusted p-values for multiple comparisons to control the FDR, are provided alongside p-values to highlight significant deviations.

Diagnosis	Diagnosis code explained	n	$\tilde{x}$	s	p-value	q-value∗
N18	Chronic kidney disease	3006	4.415	0.719	5.00E-107	2.63E-104
N17	Acute renal failure	2026	4.385	0.819	7.84E-45	2.06E-42
I10	Essential (primary) hypertension	6761	4.072	0.442	3.02E-34	5.30E-32
E11	Type 2 diabetes mellitus	3110	4.243	0.557	7.90E-30	1.04E-27
I70	Atherosclerosis	1674	4.267	0.545	1.11E-23	1.16E-21
I63	Cerebral infarction	1498	4.031	0.426	1.16E-20	1.02E-18
I50	Heart failure	2822	4.224	0.557	4.69E-19	3.52E-17
T86	Complications of transplanted tissue	285	4.497	0.688	4.43E-17	2.91E-15
I42	Cardiomyopathy	915	4.277	0.513	6.89E-17	4.02E-15
R47	Speech disturbances, not elsewhere classified	1049	4.028	0.413	1.35E-16	7.09E-15
Z94	Presence of transplanted organs and tissues	621	4.335	0.606	4.09E-16	1.96E-14
I79	Disorders of arteries, arterioles, and capillaries	279	4.492	0.712	1.84E-15	8.07E-14
G63	Polyneuropathy in diseases	309	4.454	0.716	4.89E-14	1.98E-12
Z95	Presence of cardiac & vascular implants & grafts	4033	4.185	0.459	4.77E-13	1.79E-11
C71	Malignant neoplasm of brain	297	3.984	0.348	9.80E-13	3.43E-11
G81	Hemiplegia	964	4.042	0.401	1.12E-12	3.68E-11
U50	Motor Function Impairment	514	4.006	0.402	1.54E-12	4.75E-11
H81	Disorders of vestibular function	108	3.880	0.354	2.00E-11	5.84E-10
R11	Nausea and vomiting	262	3.943	0.453	5.28E-11	1.46E-09
K35	Acute appendicitis	94	3.835	0.393	5.91E-11	1.55E-09

^a^q-value established by correcting the p-values from multiple t-testing.

### 3.3 Reference intervals

The removal of significantly differing values allowed for the RIs to reflect variations across sex and age-stratified reference populations more accurately. The most apparent changes in the position and width of inferred RIs were seen when individual ICD-10 code associations were grouped based on co-occurrences of ICD-10-GM codes and subsequently removed (refer to Supplementary Table 1). This process, known as DD with Clustering, involved hierarchical clustering using 800 clusters, utilizing a bottom-up approach. This technique significantly reduced the number of test results across most strata, resulting in notably wider CIs compared to those derived without clustering. The observed differences amongst most sex and age strata were more pronounced in the DD with Clustering than without Clustering (Table 2). The DD with clustering also made the reference intervals wider for female patients, ages 20–49, compared to the male patient in similar ages.

**Table 2:**
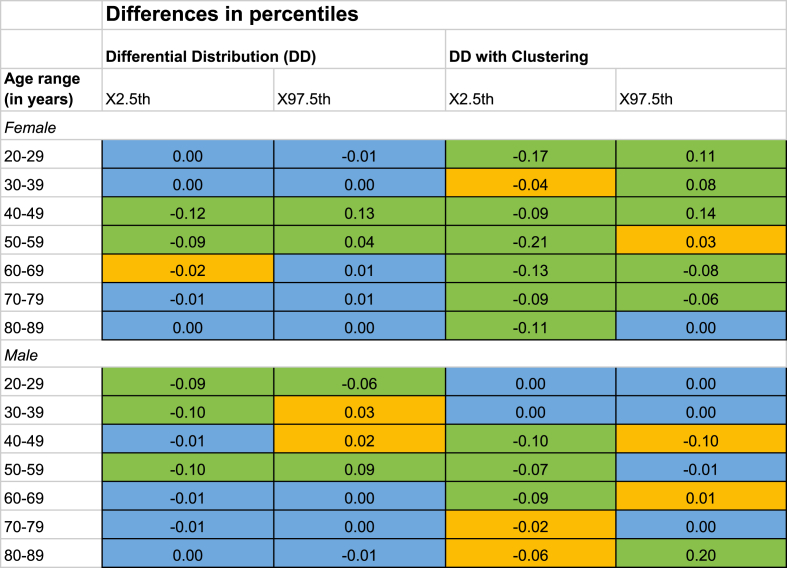
Potassium X_2.5th_ and X_97.5th_ percentiles differences (mmol/L) in Differential Distributions with and without clustering, compared to the Global Distribution. The changes are color-coded: Green for non-overlapping confidence intervals (CIs) between the percentiles, yellow for overlapping CI, and blue for non-significant differences between the percentiles from the GD's CIs.

## 4 Discussion

### 4.1 The Differential Distribution Method for potassium results

The initial analysis of the GDs, which included all available test results, revealed substantial variances. The study population exhibited a nearly balanced sex distribution, yet a significant proportion of elderly patients (over the age of 60) as typically seen in a tertiary hospital setting (Fig. 3). The DDM effectively removed a higher number of test results from these older patient groups across both sexes, reflecting the increased prevalence of multimorbidity among the elderly [12]. The wide range of reasons for patient admissions necessitates a cautious approach to data interpretation, especially for RI inference. It is essential to differentiate between ICD-10 diagnosis codes that mostly yield pathological test results, which could skew the accuracy of inferred RIs. In the specific analysis for males, ages 60–69, prevalent ICD-10 codes included those for kidney disorders (N17, N18) and heart insufficiencies (I50), aligning with the established approach to remove these results *a priori* [5]. It is noteworthy that ICD-10 codes referring to systemic diseases (E11), essential primary hypertension (I10), heart-related conditions (I42) also appear in the provided exemplary slice. This can be expected as i.e. hypertension is frequently treated with diuretics, affecting blood potassium levels [27]. Potassium potentially can be a good diagnostic marker for detecting heart diseases [28]. Considering the range of diagnoses during the diagnostic process is not only clinically relevant, but also crucial for accurate RI inference using indirect methods. Unlike other indirect methods that exclude results based on the department of origin (e.g., oncology or intensive care units), the DDM focuses on excluding only those "pathological values" identified through ICD-10 codes during the patient data stratification process. This approach allows for the formation of adjusted reference populations that more accurately reflect the diversity of the locally admitted patient population. Moreover, the DDM sheds light on which ICD-10 subpopulations of test results significantly skew the GD and contribute to the tails of the distributions. The inclusion of both Student's t-testing and Mann-Whitney-U testing (when normality assumptions are not met) allows for the comparison of analytes that initially present non-Gaussian distributions. However, in the subsequent iterative RI inference step, the DDM assumes convergence towards an underlying Gaussian distribution. As a limitation, analytes with distributions that strongly deviate from the Gaussian normal distribution may be less compatible with this final inference step.

### 4.2 Clustering of ICD-10 codes

While ICD-10 codes are commonly used to classify and detect comorbidities [29,30], their use in clustering for RI inference is novel. The clustering step in the DDM introduces an innovative way to investigate the comorbidities inherent in the presented study populations. Grouping the test results by clustering their associated ICD-10 codes *(Differential Distribution with Clustering*) removed more test results from the GD compared to separately grouping and then comparing test results by their ICD-10 codes *(Differential Distribution*) did. This clustering, based on the co-occurrence of diagnoses, formed groups of ICD-10-GM codes that reflect common clinical patterns. However, this approach has limitations, as these codes are often optimized for billing rather than research. Notably, in some slices the clustering resulted in singular clusters, highlighting the fact that certain diagnoses already contribute significantly differing test results on their own. However, removing results that are grouped individually without the clustering was seemingly the more conservative removal method, especially visible in the elderly patient strata for both sexes. While still in its formative stages, the implementation of the clustering technique to form ICD-10-GM code groups shows promise to improve the performance of the DDM. The method's approach has certain limitations, as the selection of cluster numbers during hierarchical clustering requires tangible input from the clinical diagnostic practice. Further research should explore how to integrate clinical context from the outset, potentially improving the relevance of clusters in clinical decision-making.

### 4.3 Estimated reference intervals

The considerable variance in the potassium test results from the GD contributed to the considerable width of the estimated RIs. Despite this, the estimated RIs still exhibited variability across the age and sex strata, highlighting the necessity for age- and sex-adjusted RIs (Supplementary Table 1). By removing significantly differing test results, grouped by their individual ICD-10 code or clusters of ICD-10 codes, the observed test result variance was reduced as a direct and -ostensibly- trivial consequence, while the variability of RIs across the strata remained. Other approaches towards the goal of reducing test result variance may include winsorization of estimated parameters using a moving window approach or other robust statistical estimation techniques [31]. In our approach, we have relied on the massive dataset available which conveniently allows for different options that most probably yield similar results. Our proposed approach involved removing significantly differing test results based on their individual ICD-10 code or clusters of ICD-10 codes, in essence providing a more informed step when refining the reference population from which RIs are finally inferred. This strength, however comes with a trade-off, as our approach requires large sample sizes to enable reliable RI inference. In line with other indirect methods, approximately 5000 test results are needed per mixed patient stratum to provide reliable RI estimates, particularly in populations with increased proportions of pathological values [32]. This implies a minimum of around 70000 test results, when stratifying by sex and by the 10-year age slices. To adequately capture the diversity of comorbidities encountered in routine clinical populations, an expanded cohort size of 100000 test results is recommended when applying the DDM in heterogeneous patient populations.

It is important to recognize the dual clinical applications of the RIs generated by the DDM. Firstly, the observed reduction in variances in all strata suggests a decrease in non-pathological test results, leading to more homogeneous reference populations. When considering patient test results from routine monitoring, most data is reflective of the spectrum of diseases presented, however a substantial volume of non-pathological values is generated as well. This allows the generation of reference target ranges from contaminated data sources that still reflect the local patient population. Secondly, for specific diseases or combinations thereof, the DDM can establish “disease-related” expectation ranges. These ranges serve as a comparative tool, aligning a patient's results with those of similar "digital twins" within the database, thus offering a more precise diagnostic assessment. These ranges could help to adjust the commonly used RIs to the various multimorbidities present in the local population, offering a more nuanced comparison tool for clinical diagnostics. In contrast to personalized RIs, which are predominantly shaped by the within-subject variation [32], the population-based RI derived from the DDM are designed to reflect the broader between-subject variation inherent within the local clinical setting. The need to include the health condition within the diagnostic process is evident, and providing the adjusted expectation ranges in addition to the standard RIs can presumably add more diagnostic value. It is worth emphasizing that there is a trade-off between choosing smaller age ranges for the stratification vs the resulting sample sizes. Moreover, adopting a sliding age-range stratification with varying window sizes could allow for the development of quasi-continuous RIs, further refining diagnostic accuracy across diverse patient groups, e.g., newborns, children, or elderly patients.

## 5 Conclusion

The study demonstrates the use of a novel RI mining approach for extracting insights from routine clinical data. In addition to considering patients' age and sex, the DDM considers the patients’ health condition directly. This has previously not been considered at the level of each included test result. The variety of patients admitted to a tertiary hospital is diverse, encompassing a wide range of diseases. Explicit removal of patient test results that stem from significantly differing distributions grouped by their ICD-10 codes reduced the variance observed in the sex- and age-stratified reference population, aligning them for the inference of adjusted reference intervals. Additionally, the clustering of ICD-10 codes proved particularly effective in older patients strata, addressing the higher prevalence of multimorbidities commonly present in said strata. With the DDM, we demonstrate that considering the health status of patients in addition to their age and sex, can significantly improve the relevance and applicability of RIs in clinical practice, leading to more informed and effective patient care.

## CRediT authorship contribution statement

**David Schär:** Writing – review & editing, Writing – original draft, Visualization, Software, Methodology, Investigation, Formal analysis, Conceptualization. **Tobias U. Blatter:** Writing – review & editing, Writing – original draft, Visualization, Validation, Software, Methodology, Investigation, Formal analysis, Conceptualization. **Harald Witte:** Writing – review & editing, Visualization, Validation, Software, Project administration, Data curation. **Jivko Stoyanov:** Writing – review & editing, Validation, Funding acquisition. **Martin Hersberger:** Writing – review & editing, Validation, Funding acquisition. **Christos T. Nakas:** Writing – review & editing, Visualization, Validation, Supervision, Software, Data curation. **Alexander B. Leichtle:** Writing – review & editing, Writing – original draft, Supervision, Software, Methodology, Funding acquisition, Conceptualization.

## Ethics approval and consent to participate

Due to the use of anonymized data, this study received an ethics waiver from the cantonal ethics committee of Bern (Business Administration System for Ethics Committees, waiver no. 2020-00630). Informed consent to participate is not applicable for this study by the same waiver no. 2020-00630.

## Data statement

The datasets analyzed during this study are not publicly available as they are subject to compliance with the General Data Protection Regulation (GDPR) but are available on reasonable request and conditional on ethics committee clearance and subject to agreement from the original data provider.

The developed application is available for download and use with the users’ own clinical cohort data.

Project name: Differential Distribution App (DDA)

Project home page: https://github.com/Computational-Medicine-Group/DDA.

Operating system: Platform independent.

Programming language: R version≥4.1.2, RStudio version≥2021.09.2.

R packages dependencies: dplyr≥1.1.0, plotly≥4.10.1, data.table≥1.14, reshape2≥1.4.4, cowplot≥1.1.1, lsa≥0.73.3, word2vec≥0.3.4, text2vec≥0.6.3, matrix≥1.5–3, mclust≥6.0.0, infotheo≥1.2.0.1, pheatmap≥1.0.12, qvalue≥2.26.0, scrutiny≥0.2.4, shiny≥1.7.4, shinyWidgets≥0.7.

For academic use, the app is licensed under the MIT license. Restrictions apply for commercial/non-academic use under a separate license.

## Funding sources

This research has been funded by the Swiss Personalized Health Network (SPHN) grant number 2018DEV22.

## Declaration of competing interest

The authors declare the following financial interests/personal relationships which may be considered as potential competing interests.

Reports a relationship with that includes:. Has patent pending to. If there are other authors, they declare that they have no known competing financial interests or personal relationships that could have appeared to influence the work reported in this paper.

## Figures and Tables

**Table:** 

RI(s)	Reference Interval(s)
DDM	Differential Distribution Method
DD	Differential Distribution
GD	Global Distribution
ICD-10 system	International Classification of Diseases coding system, Tenth Edition
NLP	Natural Language Processing
GUI	Graphical User Interface
GM	German Modification
FDR	False Discovery Rate
CI(s)	Confidence Interval(s)

## Data Availability

The authors do not have permission to share data.
