# Supplementary material for: Differential Distributions: A refined methodology to indirect reference interval estimation by including Patient's health status according to associated ICD-10 codes
Source: Pract Lab Med. 2025 Jul 9;46:e00492. doi: 10.1016/j.plabm.2025.e00492 (PMC12275890; doi:10.1016/j.plabm.2025.e00492)
Supplement: Multimedia component 1 [file mmc1.docx]

**Supplementary Table 1**: **Potassium X_2.5th_ and X_97.5th_ percentiles (mmol/L) inferred by the use of the iterative method from the three different distributions.** Reference interval estimates are drawn from the Global Distribution (Left), the Differential Distribution (Middle) and Differential Distribution generated using hierarchical clustering with 800 clusters (Right). The RIs also include the 90% confidence intervals (in the bracket), providing insight into their respective precision.

|  | **Global Distribution** | | | **Differential Distribution** | | | **Differential Distribution with Clustering** | | |
| --- | --- | --- | --- | --- | --- | --- | --- | --- | --- |
| **Age range**  **(in years)** | **N** | **X2.5th** | **X97.5th** | **N** | **X2.5th** | **X97.5th** | **N** | **X2.5th** | **X97.5th** |
| *Female* | | | | | | | | | |
| 20-29 | 9829 | 3.21 (3.2-3.22) | 4.41 (4.4-4.42) | 5807 | 3.21 (3.19-3.22) | 4.4 (4.39-4.42) | 347 | 3.04 (2.97-3.1) | 4.52 (4.46-4.58) |
| 30-39 | 14674 | 3.22 (3.22-3.23) | 4.42 (4.41-4.42) | 8936 | 3.22 (3.21-3.23) | 4.42 (4.41-4.43) | 710 | 3.18 (3.14-3.22) | 4.5 (4.46-4.54) |
| 40-49 | 11818 | 3.28 (3.27-3.29) | 4.5 (4.49-4.51) | 4198 | 3.16 (3.14-3.18) | 4.63 (4.62-4.65) | 345 | 3.19 (3.13-3.25) | 4.64 (4.58-4.7) |
| 50-59 | 17126 | 3.26 (3.25-3.27) | 4.62 (4.61-4.63) | 8117 | 3.17 (3.16-3.18) | 4.66 (4.65-4.67) | 481 | 3.05 (3-3.11) | 4.65 (4.59-4.7) |
| 60-69 | 21122 | 3.17 (3.16-3.17) | 4.74 (4.74-4.75) | 15955 | 3.15 (3.14-3.16) | 4.75 (4.74-4.76) | 1138 | 3.04 (3-3.08) | 4.66 (4.63-4.7) |
| 70-79 | 27484 | 3.13 (3.12-3.14) | 4.85 (4.84-4.86) | 24714 | 3.12 (3.12-3.13) | 4.86 (4.85-4.86) | 3903 | 3.04 (3.02-3.07) | 4.79 (4.77-4.81) |
| 80-89 | 23935 | 3.12 (3.11-3.13) | 4.9 (4.89-4.91) | 21475 | 3.12 (3.11-3.13) | 4.9 (4.89-4.91) | 4361 | 3.01 (2.98-3.03) | 4.9 (4.88-4.92) |
| *Male* | | | | | | | | | |
| 20-29 | 7090 | 3.28 (3.27-3.29) | 4.59 (4.58-4.6) | 1687 | 3.19 (3.16-3.21) | 4.53 (4.5-4.56) | 7090 | 3.28 (3.27-3.29) | 4.59 (4.58-4.6) |
| 30-39 | 8933 | 3.28 (3.27-3.29) | 4.61 (4.6-4.62) | 3443 | 3.18 (3.16-3.2) | 4.64 (4.62-4.66) | 8933 | 3.28 (3.27-3.29) | 4.61 (4.6-4.62) |
| 40-49 | 14124 | 3.28 (3.27-3.29) | 4.71 (4.7-4.72) | 7364 | 3.27 (3.26-3.29) | 4.73 (4.71-4.74) | 169 | 3.18 (3.09-3.26) | 4.61 (4.53-4.7) |
| 50-59 | 27155 | 3.36 (3.36-3.37) | 4.73 (4.72-4.74) | 21608 | 3.26 (3.26-3.27) | 4.82 (4.82-4.83) | 1850 | 3.29 (3.27-3.32) | 4.72 (4.69-4.75) |
| 60-69 | 37210 | 3.35 (3.34-3.35) | 4.83 (4.82-4.83) | 33829 | 3.34 (3.34-3.35) | 4.83 (4.82-4.84) | 2740 | 3.26 (3.24-3.29) | 4.84 (4.82-4.87) |
| 70-79 | 39882 | 3.26 (3.25-3.26) | 4.95 (4.94-4.96) | 37136 | 3.25 (3.24-3.26) | 4.95 (4.95-4.96) | 4764 | 3.24 (3.22-3.26) | 4.95 (4.93-4.97) |
| 80-89 | 21955 | 3.24 (3.23-3.25) | 4.99 (4.98-5) | 20361 | 3.24 (3.23-3.25) | 4.98 (4.97-4.99) | 1256 | 3.18 (3.14-3.23) | 5.19 (5.14-5.23) |
